# Immunization Coverage, Equity, and Access for Children with Disabilities: A Scoping Review of Challenges, Strategies, and Lessons Learned to Reduce the Number of Zero-Dose Children

**DOI:** 10.3390/vaccines13040377

**Published:** 2025-03-31

**Authors:** Godfrey Musuka, Diego F. Cuadros, F. DeWolfe Miller, Zindoga Mukandavire, Tapiwa Dhliwayo, Patrick Gad Iradukunda, Oscar Mano, Tafadzwa Dzinamarira

**Affiliations:** 1 International Initiative for Impact Evaluation, Harare P.O. Box 0002, Zimbabwe; 2 Digital Epidemiology Laboratory, Digital Futures, University of Cincinnati, Cincinnati, OH 45221, USA; 3 Department of Tropical Medicine and Medical Microbiology and Pharmacology, University of Hawaii at Manoa, Honolulu, HI 96822, USA; 4 Institute of Applied Research and Technology, Emirates Aviation University, Dubai P.O. Box 53044, United Arab Emirates; 5 Department of Community Medicine, Midlands State University, Gweru P.O. Box 9055, Zimbabwe; dhliwayot@staff.msu.ac.zw; 6 Rwanda Food and Drug Authority, Kigali P.O. Box 3243, Rwanda; gadpatrickiradukunda@gmail.com; 7 Department of Public Health, University of the Western Cape, Robert Sobukwe Road, Bellville 7535, South Africa; 8 School of Health Systems and Public Health, University of Pretoria, Pretoria 0002, South Africa; 9 ICAP in Zimbabwe, Harare P.O. Box 263, Zimbabwe

**Keywords:** immunization equity, children with disabilities, low- and middle-income countries, vaccine barriers, vaccination strategies, inclusive healthcare

## Abstract

**Background**: Children with disabilities, particularly in low- and middle-income countries (LMICs), face heightened risks of vaccine-preventable diseases due to a range of systemic and social barriers. Although immunization is a fundamental human right and a proven public health intervention, this vulnerable group is often overlooked in policy and practice. Understanding the factors compromising vaccine equity for these children is critical to reducing zero-dose prevalence and improving health outcomes. **Methods**: This scoping review examined peer-reviewed, gray literature from 2010 to 2024. Searches were conducted in PubMed, Google Scholar, and relevant organizational reports (WHO, UNICEF). Studies addressing children with disabilities and focusing on immunization barriers, interventions, or lessons learned were selected. English-language publications were screened in title/abstract and full-text stages. Key data extracted included population, barriers, and immunization outcomes. Since this review focused on articles in English, this is a key limitation. Results were synthesized thematically to identify recurring patterns and to guide improved interventions and policies. **Results**: Twelve articles met the inclusion criteria. Key barriers identified were inadequate healthcare infrastructure, insufficient provider training, limited follow-up services in rural regions, societal stigma, and pervasive misconceptions around both disability and vaccines. Factors such as maternal education, logistical support for caregivers, and using low-sensory, inclusive vaccination settings were consistently linked with better outcomes. Effective strategies included mobile vaccination units, tailored interventions (e.g., distraction or sedation techniques), school-based immunization programs, and robust community engagement to address stigma. Lessons learned underscored the importance of flexible, individualized care plans and empowering families through transparent communication. **Conclusions**: Children with disabilities continue to experience significant gaps in immunization coverage, driven by intersecting barriers at the individual, health system, and societal levels. Scaling tailored interventions, inclusive policies, strengthened infrastructure, and ongoing research can help ensure these children receive equitable access to life-saving vaccinations.

## 1. Introduction

Immunization is widely recognized as one of the most effective public health interventions to prevent infectious diseases, particularly among children [1]. Vaccines have been critical in reducing mortality and morbidity from vaccine-preventable diseases, significantly improving global health outcomes. However, achieving high immunization coverage remains a challenge, especially in low- and middle-income countries (LMICs) [2], where barriers such as geographical remoteness, poverty, limited healthcare infrastructure, and resource constraints hinder access to life-saving vaccines [3]. Within these populations, certain groups face even more significant obstacles, including children with disabilities, who are often excluded from mainstream health services due to systemic gaps and societal stigma [4,5,6].

Children with disabilities face significant barriers to accessing healthcare, including immunization, due to physical, environmental, and cultural challenges. In LMICs, these children are less likely to receive timely vaccinations, partly because of inadequate healthcare facilities, a lack of disability-inclusive services, logistics, and societal discrimination. For example, Thota et al. revealed that research on improving access to healthcare for children with disabilities in LMICs is lacking in several critical areas, including the provision of general health services and the accessibility of healthcare settings [7]. Their evidence and gap mapping highlighted the need for more effective interventions in areas such as non-discrimination, tackling harmful stereotypes, and ensuring access to essential services like water, sanitation, and hygiene [7]. The study also identified gaps in policies and programs that promote inclusion and participation, which are essential for ensuring that children with disabilities can fully enjoy their rights, including the right to health, as outlined in international frameworks like the Convention on the Rights of the Child (CRC). Immunization, as a fundamental human right, must be accessible to all children, including those with disabilities, to protect them from preventable diseases and reduce their disproportionate health risks.

Numerous studies reveal significant gaps in immunization coverage between children with disabilities and their non-disabled peers, even in countries with robust health systems and strong immunization programs. For example, in the UK, Emerson et al. found that children with intellectual disabilities were less likely to be fully vaccinated compared to their peers without disabilities [8]. The study, which analyzed data from the Millennium Cohort Study, showed that vaccination rates for children with intellectual disabilities were lower at the ages of nine months and three years, with these children being more than twice as likely to be non-vaccinated compared to non-disabled children [8]. This disparity persisted despite adjustments for socioeconomic factors. Similarly, O’Neill et al.’s review highlighted that 78% of studies across various countries, including the USA and the UK, found lower immunization uptake in children with disabilities [6]. The review emphasized that these vulnerabilities were often linked to various factors, including inadequate access to healthcare services, logistical barriers, and the broad and inconsistent definitions of disability used in different studies. For example, while some studies from the USA suggested no significant difference in vaccine uptake among children with special healthcare needs, other studies found that specific populations, such as children with spina bifida or autism, had notably lower vaccination rates [6]. These disparities, occurring even in high-income countries with established immunization programs, underscore the urgency of addressing this challenge, specifically in LMICs, where healthcare access and systems may be even more constrained.

This scoping review examined the factors contributing to disparities in immunization coverage for children with disabilities. We also aimed to identify effective strategies for designing and prioritizing immunization interventions to ensure that children with disabilities are included, aiming to reduce zero-dose children and reach underserved communities in LMICs. Additionally, the review assessed key lessons learned from past initiatives in delivering immunization programs to children with disabilities in LMICs.

## 2. Methods

### 2.1. Study Design

A scoping review of the available literature was conducted. The review was guided by the Preferred Reporting Items for Systematic Reviews and Meta-Analysis Protocols (PRISMA-P) guidelines [9] and aligned with the Centre for Reviews and Dissemination (CRD) guidance for undertaking systematic reviews in healthcare [10].

### 2.2. Research Question and Study Eligibility

The population–concept–context (PCC) framework (Table 1) was used to set the eligibility criteria for the review question following recommendations from the Joanna Briggs Institute [11].

This review addresses the two research questions outlined below.

1. What are the key barriers and systemic factors contributing to inequities in immunization coverage for children with disabilities globally?

2. What strategies have been identified or implemented to ensure children with disabilities are not left out by immunization programs?

### 2.3. Literature Sources

A search was conducted on the PubMed and Google Scholar electronic databases. Additional sources, such as grey literature and reports from WHO, UNICEF, and other relevant international organizations, were also consulted for relevant publications reporting routine immunization service delivery among children with disabilities.

### 2.4. Inclusion Criteria

This review included peer-reviewed publications on immunization access for children with disabilities published between 2010 and 2024. To be eligible, articles were required to focus on either barriers to immunization or strategies for improving access for children with disabilities. Only articles published in English were considered eligible. Studies were excluded if they did not address children with disabilities or did not relate to immunization access.

To address the varying definitions of disability across studies, this scoping review adopted the World Health Organization’s International Classification of Functioning, Disability, and Health (ICF) as its conceptual framework [12,13]. The ICF defines disability as an umbrella term encompassing impairments, activity limitations, and participation restrictions, where a health condition interacts with personal and environmental factors. In line with this holistic view, we included studies focusing on a broad range of physical, sensory, intellectual, and developmental disabilities. This approach is intended to capture the diverse experiences and needs of children who may face barriers to immunization due to their functional limitations or social/environmental constraints.

#### 2.4.1. Operational Criteria

Physical disabilities: Conditions significantly limiting mobility or motor functions (e.g., cerebral palsy).

Sensory disabilities: Vision or hearing impairments that require specific accommodations (e.g., Braille materials, sign language interpretation).

Intellectual disabilities: Diagnosed conditions affecting cognitive functioning, such as Down syndrome or global developmental delay.

Developmental disabilities: Neurodevelopmental conditions like autism spectrum disorder (ASD), attention deficit hyperactivity disorder (ADHD), or other pervasive developmental delays.

#### 2.4.2. Handling Varied Definitions During Screening

Inclusive Screening: We used broad search terms (e.g., “children with disabilities”, “special healthcare needs”, “neurodevelopmental disorders”, “physical impairment”) to capture studies even if they employed alternative or narrower definitions of disability.

Cross-Checking Against ICF: In cases where authors used non-standardized definitions (e.g., “children with learning difficulties” or “children with special needs”), we compared these descriptions against the ICF framework to verify whether the study population fit our overall criteria of functional impairment and potential participation restrictions.

Contextual Judgement: When an article’s definition of disability was ambiguous, we reviewed the detailed methods and population characteristics to determine alignment with the ICF perspective. If the study population overlapped substantially with the recognized categories of disability outlined above, the article was retained.

#### 2.4.3. Rationale

By grounding our inclusion criteria in the ICF, we aimed to avoid overly restrictive or overly broad definitions of disability, thus maximizing relevance and comparability among included studies. This ensures we capture the multifaceted nature of disability (biological, psychological, social) and retain studies that reflect the diverse obstacles and facilitators to immunization.

This clear operationalization allows for a consistent interpretation of “children with disabilities” and mitigates the risk of excluding relevant subgroups who might not neatly fit a single diagnostic label. It also promotes transparency in how we approached articles that used differing or less-formalized disability definitions.

### 2.5. Search Strategy

The search strategy was co-developed and pilot-tested with the assistance of a senior health science librarian and pediatrician. The search strategy accounted for the number of terms used to describe disability, incorporating broad descriptors and specific diagnoses to ensure a comprehensive search of disability literature. The search terms included a combination of keywords and medical subject headings (MeSH), such as “children with disabilities”, “immunization”, “health equity”, “vaccination access”, and “zero-dose children”. The strategy was tailored to each database to ensure comprehensive retrieval of relevant studies. The search dates included 2010 to 2024.

### 2.6. Title, Abstract, and Full-Text Screening

Two independent reviewers screened titles, abstracts, and full-text articles for eligibility. Studies identified through database searches were exported to EndNote [14]. Duplicate articles were removed. Studies were then exported from EndNote to the Covidence systematic review management platform [15]. This desk review followed the PRISMA-P guidelines.

### 2.7. Data Abstraction and Analysis

A customized data extraction form was developed to systematically collect information from each eligible study. Extracted data points included author(s), publication year, study design, study location, population characteristics, barriers identified, strategies implemented, key findings, and conclusions. The extracted data were analyzed thematically to identify common patterns and recurring themes related to immunization coverage and access for children with disabilities. These themes were then categorized under the PCC framework to better understand the interplay between systemic barriers, strategies for improvement, and the context of immunization programs. The analysis also considered lessons learned from past initiatives, focusing on actionable insights that can inform future interventions to improve immunization equity for children with disabilities.

### 2.8. Justification of Exclusion Criteria

The exclusion of 102 articles during the screening process was based on rigorous criteria to ensure relevance and quality. At the abstract level, studies were excluded if they did not specifically address immunization for children with disabilities or if the focus was unrelated to barriers, strategies, or lessons learned in improving vaccine access. For example, articles discussing general healthcare systems without mentioning vaccination programs or disability-inclusive practices were excluded. At the full-text level, studies were excluded if they lacked primary data, provided only anecdotal evidence, or did not meet quality appraisal benchmarks, such as unclear methodologies or small, unrepresentative sample sizes. For instance, a study focusing on adult vaccination programs with a brief mention of childhood immunization was excluded due to insufficient relevance. These exclusion criteria were applied consistently to maintain the integrity and focus of the review.

## 3. Results

### 3.1. Characteristics of Included Studies

**Figure 1:**
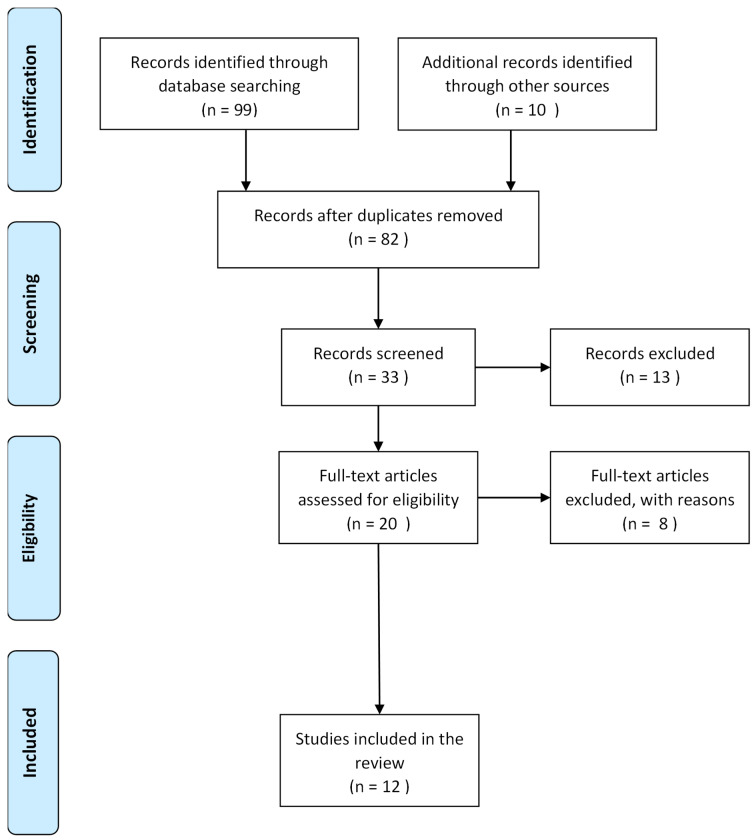
PRISMA flow chart of the selection of studies included in the analysis.

Our search retrieved 109 articles. After removing duplicates, 82 articles remained. Upon screening, 49 articles were removed at the title screening stage, 13 at the abstract screening stage, and another eight at the full-text screening stage, leaving 12 articles for inclusion in this scoping review [16,17,18,19,20,21,22,23,24,25,26,27] (Figure 1). Table 2 provides more details on the characteristics of the included articles. The data were synthesized into three themes, and Table 3 provides more details.

### 3.2. Barriers and Systemic Factors That Contribute to Inequities in Immunization Coverage for Children with Disabilities

The findings from the studies on immunization coverage inequities for children with disabilities were categorized into two primary themes: barriers related to healthcare systems and social factors, and the influence of education, stigma, and misconceptions. These themes reflect the multifaceted nature of inequities in vaccination access for children with disabilities and highlight systemic and societal challenges that contribute to lower immunization rates. Both themes highlight the intersectionality of healthcare accessibility and societal attitudes, which contribute to the inequities faced by children with disabilities in accessing immunization services. Our review revealed that barriers and systemic factors contributing to inequities in immunization coverage for children with disabilities include healthcare system challenges and social factors, such as the failure to obtain parental consent, logistical issues in schools with limited resources, and a lack of staff expertise in managing children’s anxiety and disruptive behavior during immunization. The absence of follow-up protocols and insufficient communication with parents further complicate the process. Additionally, geographical and financial constraints limit access to immunization services, particularly in rural or underserved areas, while humanitarian efforts often overlook the special needs of children with disabilities. Education-related factors also play a significant role, as misconceptions, such as the belief that vaccines cause autism, can lead to delayed or missed vaccinations. Stigma surrounding disabilities and the low educational attainment of caregivers, especially mothers, contribute to lower vaccination rates, as does the lack of awareness about available rehabilitation services. These systemic challenges combine to create significant barriers to equitable vaccination for children with disabilities.

A. Barriers related to healthcare systems and social factors

The first theme, barriers related to healthcare systems and social factors, is deeply rooted in the accessibility and responsiveness of health services. One key issue is the lack of resources and preparation in health facilities, which often fails to accommodate the specific needs of children with disabilities. Studies by Ong et al. (2024) [18] and Tuckerman (2024) [17] identify inadequate staff training and lack of preparation techniques as significant obstacles, particularly in LMICs with limited healthcare infrastructure. For instance, children with needle phobia, common in those with autism and ADHD, experience heightened anxiety and unsuccessful vaccination attempts, which further discourage participation in immunization programs. Additionally, the inconsistency in follow-up services, especially in rural areas, creates a barrier to maintaining vaccination schedules. Ong et al. (2024) also point out the absence of specialized services tailored for children with disabilities, which exacerbates the challenge of ensuring equitable access to immunization [18]. This systemic neglect can lead to physical and psychological barriers, such as failed vaccination attempts and the resultant avoidance of vaccination by parents and children. Furthermore, such problems as the miscategorization of symptoms, particularly in children with severe disabilities like cerebral palsy or epilepsy, contribute to lower vaccination uptake, as documented in Okoro’s study [22]. These healthcare-system-related inadequate healthcare resources and the lack of stable, accessible services in many LMICs compound barriers.

B. The influence of education, stigma, and misconceptions

The included studies underscored how societal attitudes and knowledge gaps exacerbate inequities in immunization coverage. Okoro et al. [22] highlighted that maternal education levels strongly correlate with higher vaccination rates, suggesting that a lack of knowledge about the benefits and safety of vaccines is a key driver of inequity. In many cases, parents’ limited understanding, compounded by misinformation or myths about vaccines, particularly those related to intellectual disabilities, leads to delayed or omitted vaccinations. Rosenberg (2013) discusses how parents of children with autism spectrum disorder (ASD) may delay vaccinations due to misconceptions about vaccines causing autism [19]. This mistrust is more prevalent among highly educated mothers, who may be more susceptible to misinformation. Similarly, Tuckerman (2024) identifies that parents from culturally and linguistically diverse (CALD) backgrounds may face difficulties navigating vaccination systems, resulting in a lower immunization uptake for their children with disabilities [17]. Stigma also plays a significant role, as children with more visible disabilities, such as cerebral palsy, often face social exclusion and discrimination, leading to lower vaccination rates. In comparison, children with less-visible disabilities tend to have higher vaccination rates [22]. This stigma can affect both the willingness of parents to seek vaccinations for their children and the attitude of healthcare providers toward these children.

### 3.3. Strategies That Have Been Identified or Implemented to Ensure Children with Disabilities Are Reached by Immunization Programs, and How These Can Be Scaled or Improved

**Figure 2:**
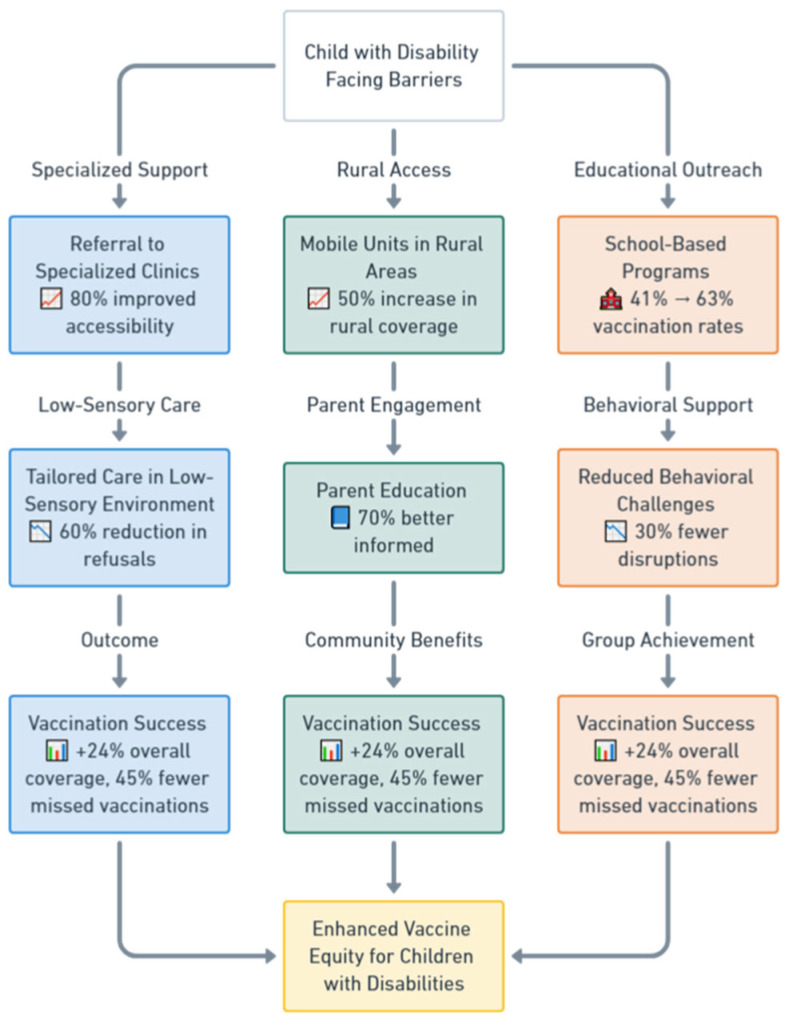
The flowchart illustrates the tailored immunization pathways for children with disabilities, starting with the central challenge, “Child with Disability Facing Barriers”. It features three main pathways: Specialized Support, Rural Access, and Educational Outreach. The Specialized Support pathway includes referrals to specialized clinics with 80% improved accessibility, leading to tailored care in low-sensory environments, which reduces vaccination refusals by 60%, and culminates in vaccination success, marked by + 24% overall coverage and 45% fewer missed vaccinations. The Rural Access pathway utilizes mobile units in rural areas, achieving a 50% increase in rural vaccination coverage, followed by parent education initiatives, where 70% of parents report being better informed, resulting in similar vaccination success outcomes. The Educational Outreach pathway employs school-based programs, improving vaccination rates in specialist schools from 41% to 63%, coupled with behavioral support that reduces disruptions by 30%, ultimately leading to vaccination success. The pathways converge on the goal of Enhanced Vaccine Equity for Children with Disabilities. Icons enhance clarity, such as a hospital symbol for specialized clinics, a book for parent education, and a school for school-based programs. Metrics, such as “80% improved accessibility” and “70% better informed”, are based on study findings. At the same time, the color coding—blue for specialized support, green for rural access, and orange for educational outreach—helps differentiate pathways visually. The outcome emphasizes significant improvements in coverage and equity for children with disabilities.

The strategies identified in the studies to improve immunization coverage for children with disabilities and reduce the number of zero-dose children were categorized into three primary themes: enhancing healthcare accessibility and service delivery, implementing tailored interventions for children with disabilities, and addressing societal factors like stigma and education (Table 4). Each theme had sub-themes that could be scaled and improved to ensure more children with disabilities are reached by immunization programs. Figure 2 illustrates a flowchart describing the tailored immunization pathways for children with disabilities.

A. Enhancing healthcare accessibility and service delivery

The first theme, enhancing healthcare accessibility and service delivery, highlights the importance of creating systems that accommodate the specific needs of children with disabilities. The included studies emphasized the need for better service infrastructure, particularly in underserved and rural areas. Tuckerman (2024) suggests that creating individualized approaches for children, including supporting parents to navigate the vaccination process, can help address gaps in follow-up services [17]. Similarly, Ong et al. (2024) highlight the value of mobile units or temporary vaccination clinics to bring services closer to families, thereby overcoming logistical barriers to accessing immunization [18]. This is complemented by Fong (2024) [20], who recommends administering vaccines in low-sensory environments to minimize anxiety and discomfort. These strategies ensure that children with disabilities are not excluded from immunization programs due to geographical, logistical, or environmental challenges.

B. Implementing tailored interventions for children with disabilities

The second theme, implementing tailored interventions for children with disabilities, focuses on making the vaccination process more manageable for children and parents. Several studies propose using distraction and sedation techniques to reduce anxiety and pain during vaccination. Ong et al. (2024) describe a “Difficult to Vaccinate” clinical pathway, which includes these strategies and ensures that children with disabilities are not excluded due to their unique needs [18]. Bray (2022) also identifies various distraction techniques, such as using handheld devices for entertainment, music therapy, or squeezing soft objects, which have proven effective in reducing anxiety [21]. Moreover, the involvement of parents in the vaccination process can significantly improve outcomes. Parental involvement, as emphasized in both Bray (2022) [21] and Okoro (2015) [22], not only provides emotional security for children but also empowers parents to make informed decisions about vaccination, such as whether to use sedatives or other techniques.

C. Addressing societal factors like stigma and education

Addressing societal factors like stigma and education, the third theme emphasizes tackling misconceptions and promoting understanding about disabilities and vaccines. Okoro (2015) suggests that healthcare workers should be trained to avoid misclassifying disabilities as contraindications to vaccination, which often leads to missed opportunities for immunization [22]. Regular health education campaigns are also recommended to dispel myths and build trust in vaccination programs. Tuckerman (2024) further underscores the need for accessible and understandable information for parents, especially those with low literacy or intellectual disabilities, to ensure they can navigate the vaccination process [17]. Additionally, addressing socioeconomic disparities, as highlighted by Okoro (2015), is crucial in ensuring equitable access to immunization for all children [22]. Policies that promote maternal education and provide financial support can help improve vaccine uptake, particularly in families with lower incomes or those from disadvantaged backgrounds [16].

### 3.4. Lessons Learned in Ensuring Equitable Access for Children with Disabilities

The included studies highlighted several vital lessons that were grouped into three key themes: creating supportive and inclusive environments, empowering families and children, and tailoring immunization services to individual needs (Table 5).

A. Creating supportive and inclusive environments

The first theme, creating supportive and inclusive environments, emphasizes ensuring vaccination settings are accessible and welcoming for children with disabilities. Tuckerman (2024) highlights that involving school staff in the vaccination process can create a supportive environment, as schools are often seen as “safe spaces” by both parents and children, making them an ideal setting for vaccinations [17]. The physical environment also plays a crucial role, as children with disabilities, particularly those with sensory sensitivities, can experience heightened anxiety during vaccinations. Creating a calm, quiet, and non-threatening space is essential to reduce this anxiety. Bray (2022) underscores the significance of offering comforting techniques, such as allowing a parent to be present or using engaging distractions like toys or music, to alleviate stress and encourage vaccination [21]. Additionally, Ong et al. (2024) stress the importance of flexible and individualized care, where the needs of the child, such as sensory sensitivities or past negative experiences, are considered when designing vaccination procedures [18]. This approach ensures that children are more likely to undergo successful vaccinations without unnecessary distress.

B. Empowering families and children

The findings of this review revealed that empowering families and children, focusing on the importance of involving parents in the vaccination process, and providing them with the tools and knowledge to advocate for their children’s needs were key to the program’s success. Ong et al. (2024) note that parental empowerment is a key strategy, where parents feel more confident and capable when they are provided with clear information and involved in decision-making, especially when it comes to the use of techniques like distraction or sedation [18]. This empowerment helps address vaccine hesitancy, as parents are better informed and more comfortable with the vaccination process. Bray (2022) also highlights the value of communication, noting that honest and transparent conversations between healthcare providers and parents are essential for building trust and reducing anxiety [21]. Parents can feel better equipped to manage their child’s healthcare needs in the long term when provided with ongoing support and addressing concerns about future vaccinations. This sense of empowerment can also extend to the children themselves, as preparing them with appropriate information and expectations can help them cope better with the vaccination process [16,18,21].

C. Tailoring immunization services to individual needs

This review also revealed that tailoring immunization services to individual needs emphasizes the necessity of adapting healthcare practices to accommodate the unique challenges faced by children with disabilities and is key to program success. Bray (2022) advocates for a neurodiversity-sensitive approach, where healthcare providers are educated about the specific needs of children with conditions such as autism or ADHD [21]. This ensures that healthcare providers respect individual differences and provide care that is not only effective but also compassionate. Tailoring immunization strategies, such as distraction techniques or providing sensory-friendly environments, can help children with neurodevelopmental disorders feel more comfortable and less anxious. Furthermore, both Tuckerman (2024) and Ong et al. (2024) point out that individualized planning, including offering multiple preparation options for both parents and children, increases the likelihood of successful vaccination [17,18]. These personalized approaches, whether through social stories or flexibility in the vaccination setting, ensure that children with disabilities are not excluded from immunization programs due to their unique needs.

## 4. Discussion

This review identifies key barriers and strategies for improving immunization coverage among children with disabilities and reducing zero-dose children, particularly in underserved and marginalized communities. The findings highlight the complex, multifaceted nature of the issue and offer valuable insights into how immunization programs can be designed and improved to ensure that children with disabilities are not excluded.

The barriers identified in this review emphasize the critical gaps in healthcare systems, social support, and societal attitudes toward disabilities. These barriers include inadequate healthcare resources, lack of staff training, and inconsistencies in healthcare service delivery, especially in rural areas. Misunderstandings about disabilities and their medical implications can lead to misclassification as vaccine contraindications, further discouraging vaccination efforts [17,22]. Furthermore, inadequate follow-up care and logistical challenges like unclear clinic locations and access issues contribute to missed vaccinations. These barriers underscore the need for comprehensive strategies at multiple health system levels. Strategies to address these barriers focus on enhancing accessibility and service delivery, particularly in underserved regions. Mobile vaccination units and strengthening infrastructure in rural areas are essential for reaching remote communities [18]. Logistical support, including clear signage and support for parents to navigate the vaccination process, is critical for reducing barriers to vaccination [21]. Moreover, creating low-sensory environments for vaccination, which alleviate anxiety for children with disabilities, is another essential strategy for improving immunization outcomes [18]. At the health facility level, offering individualized care through distraction and sedation techniques and the involvement of parents in decision-making can significantly increase vaccination uptake [21].

Designing immunization programs to reduce zero-dose children and ensure equitable access requires addressing the systemic and social factors that prevent children with disabilities from receiving vaccines [6]. The findings suggest that creating supportive and inclusive environments is key to improving immunization rates. Involving school staff in vaccination planning can help provide a safe and trusted space for both children and parents, making vaccination efforts more accessible [17]. Schools can be critical in ensuring a supportive environment, especially when parents perceive them as safe spaces [28,29]. Health systems should adapt to provide calm, non-threatening vaccination spaces, especially for children with sensory sensitivities, and use comforting techniques like toys or music to reduce anxiety [30].

This review revealed empowering families, particularly parents, as another critical strategy. Similarly, earlier work highlighted that providing parents with the knowledge and support to navigate the vaccination process, alongside involving them in decision-making regarding vaccination techniques, can reduce vaccine hesitancy and increase confidence in vaccination programs [31]. Transparent communication addresses concerns and ensures families feel supported throughout the vaccination journey [32]. The need for tailored, individualized care was a recurrent theme in this review. This includes designing personalized vaccination pathways considering each child’s medical, psychological, and developmental needs. Previous research by Leask 2006 and Helps 2019 highlighted the need for healthcare providers to receive training on neurodiversity to offer more respectful and accommodating care for children with neurodevelopmental disorders like autism or ADHD [33,34]. Tailoring immunization strategies to address the specific needs of these children is essential for improving vaccination outcomes.

### 4.1. Intersecting Stigma, Socioeconomic Disparities, and Disability

According to our review, stigma and socioeconomic disparities might intersect with disability to create compounded barriers to immunization. Stigma associated with disability often results in social exclusion, which limits access to healthcare services, including vaccination programs. Families of children with disabilities may face discrimination within their communities, further deterring them from seeking healthcare. This stigma is amplified in lower socioeconomic settings, where families may lack the financial resources to navigate systemic barriers or afford transportation to healthcare facilities. Additionally, limited parental education and cultural misconceptions about both disability and vaccines exacerbate these challenges, leading to reduced awareness of immunization benefits. For example, families living in poverty may prioritize immediate financial needs over healthcare, while stigma can prevent them from accessing community-based interventions. Addressing these intersecting issues requires a multi-faceted approach that includes anti-stigma campaigns, socioeconomic support programs, and culturally tailored community engagement initiatives. Such efforts can promote inclusive environments where families feel supported and empowered to seek immunization for their children, regardless of disability or economic status.

The intersection of disability with other identities, such as gender and ethnicity, compounds barriers to immunization access. Girls with disabilities, for instance, often face heightened discrimination due to societal biases that prioritize male children for healthcare resources in specific cultural contexts. Similarly, ethnic minority groups, such as Indigenous populations in Latin America or tribal communities in South Asia, may experience systemic exclusion from healthcare services. When combined with disability, these factors exacerbate inequities, leading to lower vaccination rates among these subgroups. Language barriers, geographic isolation, and mistrust in healthcare systems further hinder access for ethnically marginalized children with disabilities. For example, Indigenous children with disabilities may lack access to culturally tailored immunization programs, reinforcing health disparities. Addressing these intersecting barriers requires intersectional approaches that account for these populations’ unique needs and experiences, such as culturally sensitive outreach campaigns, gender-inclusive policies, and partnerships with community leaders to foster trust and inclusivity.

### 4.2. Practical Implementation, Scaling, and Evaluation of Identified Strategies

#### 4.2.1. Tailored Interventions

To implement tailored interventions, healthcare providers can adopt strategies such as individualized vaccination plans and low-sensory environments in clinics. Training workshops and simulation exercises for healthcare workers can ensure sensitivity to the needs of children with disabilities. These interventions can be scaled through partnerships with organizations specializing in disability care, enabling consistent support across multiple regions.

#### 4.2.2. Mobile Vaccination Units

Mobile units can address geographic barriers by bringing vaccines directly to underserved communities. These units should be equipped with accessibility features, such as ramps and quiet areas, and staffed by personnel trained in disability-inclusive care. Government funding and donor partnerships can help expand such programs nationally, with clear targets for reaching rural and marginalized populations.

#### 4.2.3. Community Engagement

Local leaders, disability advocacy groups, and parent associations can co-design culturally sensitive education campaigns. These initiatives should include testimonials, visual aids, and multilingual materials to combat stigma and misconceptions. Pilot programs can test effectiveness before scaling to larger communities, with adjustments based on feedback from local stakeholders.

#### 4.2.4. School-Based Vaccination Programs

Schools are ideal settings for reaching children with disabilities, particularly in specialized education environments. Policies supporting routine vaccination days, with individualized care plans and consent protocols, can streamline implementation. Scaling such programs requires collaboration between health and education ministries, alongside regular evaluations of student and caregiver satisfaction.

#### 4.2.5. Adapting High-Income Country Strategies for Low- and Middle-Income Countries

High-income countries (HICs) have successfully implemented school-based vaccination programs, digital health records, and targeted outreach campaigns to improve immunization coverage among children with disabilities. For instance, school-based vaccination in specialist education settings in Australia has shown significant success by addressing behavioral challenges and reducing access barriers [28,35]. Similarly, using electronic health records in countries like Denmark and the United States enables efficient tracking of vaccine coverage and identification of under-vaccinated groups [36,37]. However, these approaches require adaptation to fit the unique challenges of LMICs. Resource limitations, infrastructural gaps, and cultural differences necessitate cost-effective modifications, such as using mobile vaccination units to reach underserved areas and employing community health workers for targeted outreach. By adopting lessons from HICs while incorporating the local context, LMICs can develop scalable, sustainable immunization programs that address the needs of children with disabilities effectively.

#### 4.2.6. Monitoring and Evaluation (M&E)

The success of immunization programs in high-income and low- and middle-income countries underscores the importance of robust M&E frameworks. For instance, Australia’s school-based vaccination programs incorporate real-time electronic tracking systems that monitor vaccine coverage rates among students with disabilities, providing actionable data for targeted interventions [28,38]. In contrast, Uganda’s community health worker (CHW) model includes regular household surveys to assess immunization uptake and identify gaps in rural and underserved areas [39]. Metrics used in these programs include vaccination completion rates, caregiver satisfaction levels, and reductions in missed vaccination appointments. Additionally, participatory approaches, such as involving caregivers and disability advocates in program evaluations, ensure that M&E systems address the specific needs of children with disabilities. Incorporating these practices into future frameworks can enhance accountability and guide continuous improvement, particularly in resource-constrained settings.

#### 4.2.7. Funding and Policy Advocacy

Securing sustainable funding is critical for scaling. Policymakers should allocate specific budget lines for disability-inclusive immunization programs, with international agencies offering technical and financial support. Advocacy campaigns can build momentum for inclusive immunization policies, leveraging the documented benefits of these strategies to demonstrate value.

### 4.3. Limitations of the Study

A. Addressing data gaps

This review identified several critical data gaps that hinder a comprehensive understanding of immunization barriers for children with disabilities in LMICs. Notably, there is a lack of longitudinal studies that track vaccination coverage and health outcomes over time, making it difficult to assess the long-term effectiveness of interventions. Additionally, certain disability types, such as sensory impairments or rare developmental conditions, are underrepresented in the literature. This underrepresentation limits the generalizability of findings and may result in overlooked barriers or ineffective strategies for these subgroups. Furthermore, geographic disparities in research focus exist, with limited data from rural settings where systemic challenges are often more pronounced. To address these gaps, future research should prioritize inclusive study designs that encompass diverse disability types and settings, incorporate longitudinal methodologies, and focus on equity-driven outcomes to inform targeted and effective immunization policies.

Bridging the identified data gaps requires adopting innovative methodologies and forging strategic partnerships. Participatory action research (PAR) offers a promising approach, actively involving children with disabilities, their caregivers, and community stakeholders in designing and implementing studies [40]. This approach ensures that research questions and interventions are informed by lived experiences, fostering relevance and inclusivity. Additionally, longitudinal cohort studies can provide insights into the long-term effectiveness of strategies, such as tracking vaccination uptake and health outcomes across different disability types and settings. Partnerships with disability advocacy organizations, community health workers, and international agencies like WHO and UNICEF can facilitate resource sharing and capacity building, particularly in low- and middle-income countries. Leveraging technology, such as digital health records and geospatial mapping, can improve data collection, monitor immunization disparities, and identify underserved populations. By adopting these methodologies and partnerships, researchers can generate robust evidence to inform policy and practice, advancing equitable immunization coverage for all children.

B. Methodological limitations and potential biases

The reviewed studies revealed several methodological limitations that may have influenced our findings. A significant issue was the reliance on convenience sampling, which often led to unrepresentative study populations, a lack of standard error measures, and selection bias. For example, families with greater access to healthcare or higher awareness of vaccination programs may have been more likely to participate, potentially overestimating vaccination coverage and underrepresenting those facing severe barriers. Additionally, many studies relied on self-reported data from caregivers, which is prone to recall bias and social desirability bias. Caregivers might overstate vaccine uptake to align with perceived social norms or expectations, especially when vaccination campaigns are highly promoted. Furthermore, variations in data collection methods and outcome definitions across studies make it challenging to compare results or draw generalizable conclusions. Addressing these biases in future research will require rigorous sampling techniques, standardized data collection tools, and the inclusion of diverse perspectives, particularly from underrepresented disability groups (i.e., sampling design).

Since only articles published in English were reviewed, it is possible that useful articles in other languages were excluded. It is important to note that this review did not examine whether vaccines were provided free of charge or if parents had to pay for them at the health facility. Additionally, only electronically available manuscripts were included in this review.

Another notable limitation is that this review utilized a search strategy that included only two electronic databases, PubMed and Google Scholar. While these databases are widely recognized for their extensive coverage of health-related literature, limiting the search to them may have excluded relevant studies indexed in other databases. Additionally, while efforts were made to enhance the robustness of the search by including gray literature from reputable organizations such as UNICEF and the World Health Organization, some pertinent studies may have been overlooked. Future reviews could benefit from a more comprehensive search strategy that includes multiple databases and a wider range of information sources to capture the full spectrum of relevant literature. Finally, a formal assessment of the risk of bias was not conducted due to the limited number of studies retrieved. This limitation suggests that the findings should be interpreted cautiously, as unexamined biases may influence the reported outcomes.

C. Broader methodological discussion

A critique of the methodologies used in the reviewed studies reveals several notable limitations and biases within the field. Many studies relied heavily on cross-sectional designs, which provide only a snapshot of immunization barriers and outcomes. While helpful in identifying immediate issues, these designs fail to capture longitudinal trends, such as vaccine uptake changes over time or interventions’ long-term impacts. Additionally, the reliance on convenience sampling was prevalent, often resulting in unrepresentative study populations. For instance, children with severe or less-visible disabilities might have been underrepresented, as caregivers facing significant barriers may not participate in studies.

Another methodological limitation is information bias, which lies in inconsistent data collection tools and metrics. Studies varied widely in how they measured outcomes, such as vaccine coverage rates or caregiver perceptions, complicating comparisons across findings. Some studies used self-reported data from caregivers, which may introduce recall bias, while others lacked standard definitions for key concepts like “accessibility” or “barriers”.

Lastly, geographic and cultural contexts were often insufficiently addressed. Many studies conducted in LMICs lacked region-specific adaptations to account for unique barriers such as cultural stigma or infrastructural deficits. Similarly, limited participatory research approaches meant that the perspectives of children with disabilities and their families were not always incorporated into study designs or analyses. Addressing these methodological issues is crucial for developing robust, generalizable evidence for inclusive immunization programs.

D. Inconsistencies in definitions

The variability in the definition of “disability” across the included studies posed challenges for both inclusion and analysis. Some studies defined disability narrowly, focusing on specific conditions such as intellectual disabilities or autism spectrum disorders, while others used broader definitions that included physical, sensory, and developmental disabilities. This inconsistency affected the comparability of findings, as interventions effective for one subgroup may not apply to others. For example, studies targeting children with physical disabilities emphasized environmental accessibility, whereas those involving neurodevelopmental disorders often focused on behavioral support and family engagement. These differing definitions also influenced the inclusion process; studies that lacked a clear or comprehensive definition of disability were excluded to maintain methodological rigor. Addressing this variability required careful thematic analysis to synthesize findings while acknowledging the heterogeneity in the study populations and outcomes.

### 4.4. Future Research Directions

Despite the insights gained from this review, multiple knowledge gaps hinder the development of inclusive immunization strategies for children with disabilities in LMICs. Addressing these gaps requires a concerted effort to expand the scope and improve the rigor of future research:

A. Longitudinal and Rigorous Study Designs

Long-term impact: Current evidence on the effectiveness of interventions often relies on cross-sectional or short-term studies. Future studies should adopt longitudinal designs (e.g., cohort or panel studies) that track children over time, assessing how immunization uptake and caregiver attitudes evolve as children progress through various stages of the vaccination schedule.

Rigorous sampling: Many studies rely on convenience or opportunistic sampling methods, potentially excluding the most marginalized populations. Probability-based sampling and targeted outreach strategies (e.g., partnering with community health workers) can help capture more diverse participants, including those in remote rural settings.

B. Understudied Disability Types

Visual and hearing impairments: Most existing studies focus on intellectual or physical disabilities, while sensory impairments, such as blindness or deafness, remain underexplored. These populations may face unique barriers to immunization, including communication challenges, inaccessible health education materials, and stigma or misconceptions.

Complex/multiple disabilities: Children with co-occurring conditions (e.g., autism spectrum disorder plus a physical disability) often have complex healthcare needs but are rarely included in research. Future investigations should aim to capture the intersectional experiences of these children, offering a more detailed understanding of how overlapping disabilities multiply barriers to immunization.

C. Participatory and Community-Engaged Methods

Participatory action research (PAR): Engaging caregivers, children (where appropriate), community leaders, and disability advocates in designing and implementing studies can yield richer, more context-specific insights. This approach can also foster community ownership, improving the likelihood that recommended interventions are both practical and culturally acceptable.

Stakeholder involvement: Collaboration with local health authorities, schools, and NGOs specializing in disability services can ensure that interventions are feasible and sustainable within specific cultural and resource contexts.

D. Standardization of Definitions and Outcome Measures

Adoption of the WHO ICF: Building on the work of this review, future studies should use the International Classification of Functioning, ICF, to operationalize disability consistently, thereby improving comparability across different research settings.

Aligned reporting of outcomes: Standardizing immunization coverage metrics (e.g., consistently defining “fully immunized”, “zero-dose”, and “dropout”) facilitates meta-analyses and cross-study comparisons. Clear outcome reporting also supports better monitoring and evaluation of interventions over time.

By prioritizing longitudinal research, inclusive sampling, standardized definitions, and participatory methods, the field can generate a more robust evidence base to inform policy and practice. These steps will not only deepen our understanding of immunization barriers experienced by children with diverse disability types but also drive the development of targeted, equitable, and sustainable solutions in low- and middle-income contexts.

## 5. Conclusions

In conclusion, the strategies to improve immunization coverage for children with disabilities include healthcare system reforms, tailored interventions, and societal awareness campaigns. Enhancing accessibility to services, adapting vaccination procedures to meet the needs of children with disabilities, and addressing stigma and education gaps were identified as essential strategies to ensure immunization programs can be more inclusive and effective in reaching children with disabilities. Scaling these strategies requires a concerted effort to adapt healthcare systems, train healthcare providers, and engage communities in creating a supportive environment for children with disabilities.

### Call to Action for Inclusive Immunization

This study underscores the urgent need for governments, non-governmental organizations (NGOs), and researchers to prioritize inclusive immunization programs for children with disabilities. Governments should enact policies mandating disability-inclusive practices, allocate dedicated funding for tailored interventions, and incorporate accessibility metrics into national health surveys. NGOs can play a key role in implementing community-based programs, conducting caregiver education campaigns, and advocating for systemic change at local and global levels. Researchers must address data gaps by designing longitudinal studies, developing standardized definitions of disability, and ensuring the representation of under-served populations, including those with sensory impairments or living in rural areas.

Collaboration between these stakeholders is critical to scaling successful strategies, such as mobile vaccination units, low-sensory environments, and school-based programs, while promoting an evidence-based culture of continuous improvement. By taking these steps, the global health community can advance equitable vaccine access and strengthen healthcare systems to serve all children, regardless of their abilities.

## Figures and Tables

**Table 1:** Population–concept–context framework for this scoping review.

Criteria	Operational Definition
Population	Children with disabilities - Defined in line with the WHO’s International Classification of Functioning, Disability, and Health (ICF), encompassing physical, sensory, intellectual, or developmental impairments. - Includes individuals under 18 who experience functional limitations or activity restrictions due to these impairments.
Concept	Immunization coverage and equity - Refers to the availability, accessibility, acceptability, and quality of routine vaccinations. - Encompasses the overall coverage rates (e.g., fully immunized vs. zero-dose) and equity aspects (e.g., disparities by disability type, socioeconomic status, or geography). - Examines barriers, facilitators, interventions, and outcomes related to immunization uptake.
Context	- Healthcare Systems: The context includes healthcare infrastructure and systems, examining how these systems either support or hinder immunization efforts for children with disabilities. - Global Health and Disability Policy: The review takes place within the context of global health frameworks and disability inclusion policies. This includes the United Nations’ Sustainable Development Goals (particularly Goal 3: Good Health and Well-Being, and Goal 10: Reduced Inequalities). - Barriers in Low-Resource Settings: The review may also consider the challenges in low- and middle-income countries (LMICs), where children with disabilities face compounded barriers to accessing healthcare and immunization services. - Public Health and Immunization Campaigns: The broader public health context of immunization programs and campaigns aimed at increasing vaccination rates, particularly in vulnerable groups.

**Table 2:** Characteristics of studies included in the analysis.

Study Authors	Country/Region	Economic Setting	Method	Study Period	Total Sample	Population	Participants	Themes
Lin et al. (2010) [16]	Taiwan	HICs	Cross-sectional	NA	495	Primary caregivers of children and adolescents with ID	3–24 years	Theme 3
Tuckerman et al. (2024) [17]	Australia	HIC	Qualitative cross-sectional study	June and September 2022	32	Stakeholders linked to adolescents with disability in specialist schools in Victoria, Australia	Principal/Vice Principal, *n* = 6 Other school staff, *n* = 7 Council staff, *n* = 10 Parent, *n* = 7 Adolescent, *n* = 2	Themes 1, 2, and 3
Ong et al. (2023) [18]	Australia	HIC	Qualitative cross-sectional study	September 2017 to February 2020	36	Children with intellectual and developmental disability and needle fear	Persons who took the children to vaccination	Themes 1, 2, and 3
Rosenberg et al. (2013) [19]	US	HIC	Not specified	January 14 to October 15 2009	10,000 diagnosed with ASD and 20,000 direct family members	Autism spectrum disorder (ASD)	Consented affected participants between 4 and 18 years who had completed primary history questionnaires and their consented younger siblings aged above 4.5 years and born after family concern about index development (*n* = 486 families)	Theme 1
Fong et al. (2024) [20]	Canada	HIC	Cross-sectional survey	November 2022–April 2023	109	Children diagnosed with Autism spectrum disorder (ASD), intellectual disorder (ID), and/or global developmental delay (GDD) aged 6–17 years, seen through outpatient clinics at the Ron Joyce Children’s Health Centre of McMaster Children’s hospital in Hamilton	Families with children aged 6–17 years	Themes 1 and 2
Bray (2022) [21]	General	All settings	Information from expert	Not applicable	Not applicable	Children with special needs Healthy	Not applicable	Themes 1, 2, and 3
Okoro et al. (2015) [22]	Nigeria	HIC	Cross-sectional	NA	168	children with neurological disorders	6 months to 5 years	Themes 1, 2, and 3
Mactaggart et al. (2016) [24]	Cameroon and India	LMIC	Case–control study	February–April 2014	1548	People with disabilities	India: 508 cases and 337 controls Cameroon: 429 cases and 274 controls	Theme 1
UNICEF and Leonard Cheshire Disability (2020) [25]	Not applicable	Not applicable	Not applicable	Not applicable	Not applicable	People with disabilities	Not applicable	Themes 2 and 3
UNICEF (2024) [26]	Not applicable	Not applicable	Not applicable	Not applicable	Not applicable	Children with disabilities	Not applicable	Themes 1, 2, and 3
UNICEF (2013) [27]	Not applicable	Not applicable	Not applicable	Not applicable	Not applicable	Children with disabilities	Not applicable	Themes 2 and 3
UNICEF (2023) [28]	Indonesia	UMIC	Mixed-method	2022	68	Key policymakers and implementers	Government: 55 OPD: 11 Civil Society: 1 Academic Institution: 1	Themes 1, 2, and 3

Theme 1: Barriers and systemic factors that contribute to inequities in immunization coverage for children with disabilities. Theme 2: Strategies that have been identified or implemented to ensure children with disabilities are reached by immunization programs. Theme 3: Lessons learned in ensuring equitable access for children with disabilities. Abbreviations: HIC: High-income country; LMIC: Low- and middle-income country.

**Table 3:** Barriers and systemic factors that contribute to inequities in immunization coverage for children with disabilities.

S/N	Subthemes	Authors	Findings
1.1	Barriers related to healthcare systems and social factors	Tuckerman et al. (2024) [17]	Some children do not deliver consent paperwork to their parents, failing to obtain parental or guardian consent for vaccinating. Furthermore, some parents do not trust school workers to deliver immunizations, complicating the process and not providing consent. Schools may also have insufficient resources to assist vaccination efforts, which adds logistical challenges successfully. The level of comfort with disability and assistance received during immunizations significantly impact the vaccination outcome. Meeting the individualized needs of children with disabilities can be difficult, especially when staff have little expertise managing children’s anxiety and disruptive behavior during immunization. Furthermore, the lack of a defined follow-up protocol and parents’ inadequate information regarding sedation choices create further impediments. Inappropriate follow-up settings that are not parent-friendly impede good communication and care, making it difficult to guarantee a smooth and supportive immunization procedure for these children.
		Fong et al. (2024) [20]	Vaccine administration failure due to children’s behavior.
		Okoro et al. (2015) [22]	The type of chronic neurological disorder also significantly affects the immunization coverage of children.
		Mactaggart et al. (2016) [24]	Most individuals who seek care at healthcare facilities present with illness, leading to failure to accommodate the special needs of children with disabilities. This is exacerbated by the geographical and financial constraints. which limit the families’ access to essential services like immunization.
		UNICEF (2024) [26]	Humanitarian efforts predominantly fail to make provisions for the special needs of children with disabilities, preventing them from accessing essential services like vaccination campaigns that are generally held in schools or child-friendly venues.
		UNICEF (2023) [28]	Essential health services, such as immunization, are not reaching rural areas and do not take into consideration the unique needs of children with disabilities when accessing them, despite the progress that has been made in healthcare in general.
1.2	The influence of education, stigma, and misconceptions	Tuckerman et al. (2024) [17]	Concerns regarding potential HPV vaccine reactions, as well as preferences for some vaccines over others, impact vaccination follow-up and completion rates, and this is more evident among caregivers with high education status.
		Rosenberg et al. (2013) [19]	Vaccination may be delayed due to the misconceptions about vaccines causing autism.
		Mactaggart et al. (2016) [24]	There was a low level of knowledge about rehabilitation services, which impacted the access to those services.
		Fong et al. (2024) [20]	Parent’s perspective about the influence of the children‘s behavior on vaccination success.
		Bray (2022) [21]	Parents may be hesitant to vaccinate children with special needs with a history of antimicrobial allergies.
		Okoro et al. (2015) [22]	The mother’s educational attainment and father’s occupation were observed to significantly affect the immunization coverage of children with chronic neurological disorders. Children with obvious neurological deficits whose mothers have low educational attainment are at risk of low immunization coverage. Furthermore, stigma also plays a role in lowering the immunization coverage.
		UNICEF (2023) [28]	The belief in a relationship between the vaccination and autism influenced the decision of the mothers, leading to delaying or omitting some vaccinations that were supposed to be taken in early childhood.

**Table 4:** Strategies that have been identified or implemented to ensure children with disabilities are reached by immunization programs, and how can these be scaled or improved.

S/N	Subthemes	Authors	Findings
2.1	Enhancing healthcare accessibility and service delivery	Tuckerman et al. (2024) [17]	A focus is placed on adapting information specific to students with disabilities, reassuring parents about safe vaccination spaces and ensuring staff are equipped with basic knowledge to explain the vaccination process.
		Ong et al. (2023) [18]	The parents wished that services were provided closer to their residential area, which suggests that there is a need for temporally or mobile clinics.
		UNICEF and Leonard Cheshire Disability (2020) [25]	Encouragement for incorporating disability-inclusive policies into national immunization programs in order to guarantee that healthcare facilities are accessible and equipped to serve children with different disabilities.
		Fong et al. (2024) [20]	Administering the vaccine in a low-sensory environment, decreasing wait time for vaccination, and ensuring clinic staff have experience in caring for children with special needs.
		Okoro et al. (2015) [22]	Planners and implementers of immunization programs must make greater efforts to understand the sociodemographic factors affecting immunization of children in order to extend coverage to unreached children, reduce dropout rates, and build sustainable programs.
2.2	Implementing tailored interventions for children with disabilities	Tuckerman et al. (2024) [17]	On vaccination day, a safe and structured environment should be created with distraction techniques, individualized spaces, and support staff to assist students.
		Ong et al. (2023) [18]	Distraction and sedation techniques should be included in difficult to vaccinate strategies with the aim of reducing the anxiety among children with disabilities during immunization.
		Bray (2022) [21]	The American Academy of Pediatrics (AAP) recommends vaccinating children with special needs following the same CDC immunization schedule as healthy children unless medically contraindicated. However, health-care providers may consider specific physical and psychosocial challenges when immunizing children with special needs to employ various distraction technics, including but not limited to using handheld devices for entertainment, music therapy, or squeezing soft objects, as they have proven effective in reducing anxiety.
		Okoro et al. (2015) [22]	Parental involvement in the immunization program enhances the immunization program, as it helps to reduce children’s anxiety by boosting children’s security and contributing toward an informed decision process for the parents.
		UNICEF and Leonard Cheshire Disability (2020) [25]	Collaboration with local disability-focused groups and non-governmental organizations can boost the consideration of a child’s unique physical, cognitive, or sensory needs, providing a more inclusive approach to healthcare.
		UNICEF (2024) [26]	Reasonable adjustments appropriate to the age of the child with a disability need to be made to ensure that they are given physical access to services and support for psychosocial disabilities. Also, careful identification of the need for vaccination and fighting stigma that children with disabilities or their parents may face is required.
		UNICEF (2023) [28]	The child with disabilities requires special accommodations to access the health services.
2.3	Addressing societal factors like stigma and education	Tuckerman et al. (2024) [17]	Providing information and consent forms in multiple languages and simplifying communication for parents with culturally and linguistically diverse backgrounds using visuals and tailored formats. Schools are encouraged to better educate parents on the importance of vaccination through resources, information sessions, and school communication channels like newsletters.
		Okoro et al. (2015) [22]	Healthcare workers may identify some signs and symptoms as contradictions for vaccination. Measures to reduce socioeconomic disparities also need to be taken, as they influence vaccination uptake.
		UNICEF (2013) [27]	Including children with disabilities in promotion materials, like posters for immunization programs, can raise awareness and enhance the understanding of the community on the need for vaccination uptake among children with disabilities.
		UNICEF (2023) [28]	Involvement of civil society, community members, children with disabilities, and organizations advocating for people with disabilities is instrumental in ensuring that the policies are inclusive and respond to the stigma.

**Table 5:** Lessons learned in ensuring equitable access for children with disabilities.

S/N	Subthemes	Authors	Findings
3.1	Creating supportive and inclusive environments	Tuckerman et al. (2024) [17]	Children with disabilities are very sensitive to environment, and anything that goes wrong can turn things around easily. The right physical environment with adequate infrastructure and distraction toys for children has a positive impact on the day of vaccination. The necessary efforts must be implemented to ensure that vaccination is successful at the first visit, as repeating the procedure requires extra effort. Some parents request information about alternative information that is outside the knowledge of school staff.
		Ong et al. (2023) [18]	The seduction pathway needs to be implemented in case vaccination with the distraction method is unsuccessful.
		Bray (2022) [21]	Positive techniques exist to assist the pediatric provider. It is essential to consider positioning and restraining the child with special needs during immunization procedures. Rather than lying down, sitting upright during immunizations increases a child’s comfort level and sense of control.
		UNICEF and Leonard Cheshire Disability (2020) [25]	Healthcare facilities should be inclusive to take into consideration adolescents and children in the immunization program, and the message delivered for immunization need to be disability friendly to minimize the stigma that children with disabilities frequently confront.
		UNICEF (2023) [28]	The integration of immunization services for children with disabilities among the usual immunization programs that take place at school contributed to the uptake of vaccination among children with disabilities in Indonesia. Further children with disabilities need to be considered in health-sector plans and budgets at the national and sub-national levels.
3.2	Empowering families and children	Tuckerman et al. (2024) [17]	Parents need to be informed about the vaccination date and given the opportunity to attend, fostering a more inclusive and supportive immunization process. Anxiety is common, and some adolescents are scared before being vaccinated.
		Ong et al. (2023) [27]	Having a positive experience among the parents through involvement led to empowerment of these children.
		Okoro et al. (2015) [22]	There is a need to sustain regular health education aimed at emphasizing the benefits of vaccination and dispelling false concerns about the side effects of immunizations.
		Lin et al. (2010) [16]	Health income influenced the uptake level of vaccination, and sensitivity toward the families of children with disabilities is essential for ensuring that they are aware of the benefits of vaccination.
		Bray (2022) [21]	Adequate communication with clear and concise information from healthcare professionals for parents is fundamental in highlighting and convincing the parent to ensure that their child with disabilities adheres to the CDC-recommended immunization schedule.
		UNICEF (2024) [26]	Communicating the rights of children with disabilities to access health services, including immunization, is important to boost the immunization uptake.
3.3	Tailoring immunization services to individual needs	Tuckerman et al. (2024) [17]	The need for more parent information sessions about options and additional support for vaccination outside of the school program was raised by the parents. The immunization program in special schools was perceived as convenient; however, preparing students for vaccination day and catering to individual student needs were important for the vaccination outcome.
		Ong et al. (2023) [18]	Children with needle phobias were prepared through discussion to help them deal with anxiety, and this led to a good outcome. This was complimented by a mock trial with a nitrous mask to sedate the child, and the preparation of the parents through adequate information sharing was taken into consideration a priori. This shows how individual needs were employed.
		Bray (2022) [21]	Healthcare providers, like pediatric providers, need to have knowledge related to the specific needs of children with conditions such as autism to ensure that they are given sufficient information and vaccinated.
		UNICEF and Leonard Cheshire Disability (2020) [25]	Girls with disabilities are less likely to access health services in comparison to boys with disabilities; therefore, additional attention is required to tailor the immunization program to facilitate an increase in uptake in the mentioned group.
		UNICEF (2013) [27]	Immunization services for children with disabilities require special consideration to ensure their inclusiveness.
		UNICEF (2023) [28]	The MoH Regulation No. 25/2014 on Child Health Efforts covers the provision of health services for children with disabilities, whereby they may receive healthcare beyond the healthcare facilities in place, including specialized schools, inclusive education schools, households, and other institutions. In this regulation, the health centers near specialized schools are obliged to provide immunization services at those schools.

## Data Availability

No new data were created or analyzed in this study. Data sharing is not applicable to this article.
